# Regulation of Kv1.4 potassium channels by PKC and AMPK kinases 

**DOI:** 10.1080/19336950.2017.1405196

**Published:** 2017-12-22

**Authors:** Martin Nybo Andersen, Lasse Skibsbye, Arnela Saljic, Martin Zahle Larsen, Hanne Borger Rasmussen, Thomas Jespersen

**Affiliations:** Dept. of Biomedical Sciences, Faculty of Health and Medical Sciences, University of Copenhagen, Copenhagen, Denmark

**Keywords:** AMPK kinase, Kv1.4 potassium channels, ion channels, PKC kinase, kinases, K+channels, KV channels, membrane biophysics, Nedd4-1, Nedd4-2, potassium channels, voltage channels, voltage-gated ion channels

## Abstract

Over the last years extensive kinase-mediated regulation of a number of voltage-gated potassium (Kv) channels important in cardiac electrophysiology has been reported. This includes regulation of Kv1.5, Kv7.1 and Kv11.1 cell surface expression, where the kinase-mediated regulation appears to center around the ubiquitin ligase Nedd4-2. In the present study we examined whether Kv1.4, constituting the cardiac I_to,s_ current, is subject to similar regulation. In the epithelial Madin-Darby Canine Kidney (MDCK) cell line, which constitutes a highly reproducible model system for addressing membrane targeting, we find, by confocal microscopy, that Kv1.4 cell surface expression is downregulated by activation of protein kinase C (PKC) and AMP-activated protein kinase (AMPK). In contrast, manipulating the activities of phosphatidylinositol-4,5-bisphosphate 3-kinase (PI3K) and serum and glucocorticoid-regulated kinase 1 (SGK1) were without effect on channel localization. The PKC and AMPK-mediated downregulation of Kv1.4 membrane surface localization was confirmed by two-electrode voltage clamp in *Xenopus laevis* oocytes, where pharmacological activation of PKC and AMPK reduced Kv1.4 current levels. We further demonstrate that unlike related Kv channels, Kv1.4 current levels in *Xenopus laevis* oocytes are not reduced by co-expression of Nedd4-2, or the related Nedd4-1 ubiquitin ligase. In conclusion, we demonstrate that the surface expression of Kv1.4 is downregulated by the two kinases AMPK and PKC, but is unaffected by PI3K-SGK1 signaling, as well as Nedd4-1/Nedd4-2 activity. In the light of previous reports, our results demonstrate an impressive heterogeneity in the molecular pathways controlling the surface expression of highly related potassium channel subunits.

## Introduction

The voltage-gated potassium channel Kv1.4 is part of the *Shaker* Kv-channel family and contributes to the transient outward potassium current I_to,s_ involvement in the repolarization of the cardiac action potential [1]. Kv1.4 is also expressed in various neuronal cell types where it regulates neuronal excitability [2] and in pancreatic β-cells, where activation of the channel has been suggested to influence insulin secretion [3,4]. Thus, Kv1.4 channels are involved in a wide range of important physiological functions.

For Kv1.4 channels it is known that both heteromerization with other Kv1 channel subunits as well as the glycosylation status of the channel can impact channel surface expression [5,6]. However, relatively little is known in regards to kinase signaling pathways and their impact on Kv1.4 localization. Studies suggest that the Kv1.4 channel is endocytosed in response to Protein Kinase A (PKA) activation [3,7-11]. Further, several studies suggest that Protein Kinase C (PKC) exerts an inhibitory effect upon Kv1.4. In a study by Walsh *et al.* it was demonstrated that activation of PKC inhibits the I_to_ current in cardiac fibroblasts [12]. In line with this observation, Murray *et al.* reported that PKC inhibits Kv1.4 currents in a slow time-dependent manner in *Xenopus laevis* oocytes. However, the underlying mechanism for the PKC-mediated downregulation of Kv1.4 currents has not been investigated [9].

MDCK cells constitute an excellent cell system for studying trafficking of membrane proteins, such as ion channels [13-18]. The MDCK cells are, when grown to confluency, polarized cells with an apical and a basolateral membrane. The two membrane domains are separated by tight junctions. The MDCK polarization process is initiated by cell-cell contact and is mediated by calcium-dependent E-cadherins [19]. This process can be mimicked by a so-called calcium switch assay [20]. During the polarization of MDCK cells PKC is known to be activated, and a downstream target of PKC in this process is AMPK [21,22]. AMPK can activate the E3 ubiquitin ligase Nedd4-2 [23], which has been shown to lead to endocytosis of ion channels, including the cardiac potassium channels Kv1.5, Kv7.1 and Kv11.1 [15,16,24-26]. In contrast, inhibition of Nedd4-2 ubiquitylation activity by the PI3K-Sgk1 pathway promote membrane expression of Kv7.1 and Kv1.5 [14,27]. Hence, Nedd4-2 plays a central role in both PKC/AMPK and PI3K/Sgk1 regulation of several potassium channels. Among the nine members of the Nedd4/Nedd4-like family, Nedd4-2 has the highest mRNA expression in the human heart [28]. Hence, it is likely that altered activity of cardiac expressed Nedd4-2 would have a direct effect on cardiac electrophysiology.

In this study, we have investigated the mechanisms controlling Kv1.4 surface expression. We examined whether the mentioned kinase signaling pathways, previously found to regulate the surface expression of the related Kv1.5, Kv7.1 and Kv11.1 channels [15,16,24-26], impact Kv1.4 localization thereby also regulating this potassium current involved in shaping both atrial and ventricular action potentials. Interestingly, marked differences in the regulatory mechanisms controlling Kv1.4 cell surface expression, compared to our previously published observations on related potassium channel subunits, are found.

## Results

**Figure 1.**
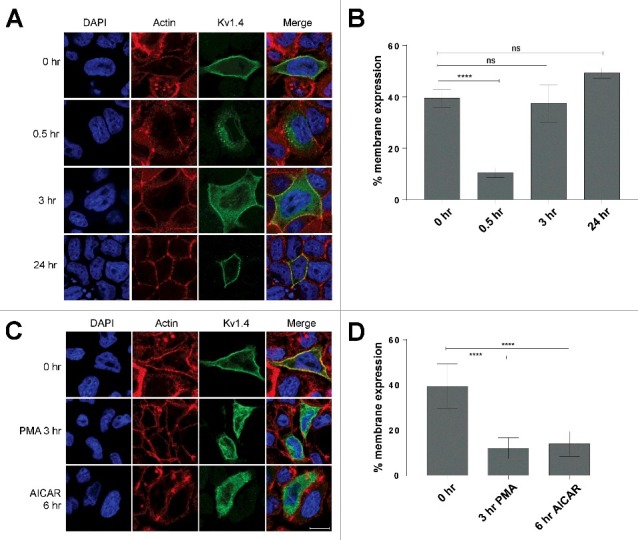
AMPK and PKC activation leads to removal of Kv1.4 channels from the cell surface. (A) MDCK cells transiently expressing Kv1.4 were subjected to a calcium switch assay (see materials and methods) to control the onset of MDCK polarization. At different time points after initiation of polarization, cells were fixed, stained with Kv1.4 antibody and subcellular localization of channels analyzed by confocal microscopy. (B) Quantification of Kv1.4 signals from (A). (C) MDCK cells transiently expressing Kv1.4 were kept unpolarized in low calcium medium. At confluency, the cells were treated with 100 nM PMA for 3 hr (to activate PKC) or 0.5 mM AICAR for 6 hr (to activate AMPK) and the subcellular localization of Kv1.4 analyzed by confocal microscopy. DAPI and phalloidin were used to stain the nucleus and actin, respectively. Scalebar for A and B, 10 µm. (D) Quantification of Kv1.4 signals from (C). The intensity of the fluorescent signal from Kv1.4 channels expressed at the plasma membrane was obtained and expressed as a percentage of the whole cell Kv1.4 fluorescent signal. All groups were statistically compared to the signal at 0 hr. Quantification was performed as described in the Materials and Methods section; *p < 0.05 and ***p < 0.001. 6 ≤ n ≤ 10 for each group. Bars represent means of each group ± SEM.

To examine whether Kv1.4 cell surface expression is regulated by kinase activity similarly to Kv1.5, Kv7.1 and Kv11.1, we used the epithelial MDCK cell line, which constitutes an excellent system for studying trafficking of membrane proteins [29]. MDCK cells constitute a highly efficient and very reproducible model system for addressing cell surface changes of membrane proteins following e.g kinase activation [30]. This system have previously been used by us and other investigators to detect changes in membrane expression of membrane proteins during kinase activation or inhibition [15,16,21,31]. In this study changes in surface expression/localization was detected by confocal microscopy. We first utilized the so-called calcium switch to polarize the cells, a process significantly impacting the cell surface expression of both Kv1.5 and Kv7.1^14-16,24^. MDCK cells were transiently transfected with Kv1.4 and the cells were subjected to the calcium switch to initiate MDCK cell polarization (see materials and methods for details). When the MDCK cells were unpolarized, Kv1.4 channels demonstrated strong membrane localization (Fig. 1A, 0 hr), but this membrane expression was significantly reduced shortly after the polarization process had begun (Fig. 1A, 0,**5** hr). The channel now showed a primarily vesicular localization pattern indicating endocytosis of surface expressed channels. 3 hours following initiation of MDCK cell polarization, Kv1.4 was found both in intracellular compartments and at the plasma membrane (Fig. 1A, 3 hr). After 24 hours the channel was primarily localized at the plasma membrane (Fig. 1A, 24 hr). Quantification of the surface versus total Kv1.4 fluorescent signals confirmed these observations. 30 minutes after initiation of MDCK cell polarization, Kv1.4 cell surface expression had significantly dropped to 11% compared to the unpolarized state. However, already 3 hours into the polarization process, Kv1.4 channels reappeared at the cell surface and 24 hours into the polarization process Kv1.4 surface expression was completely restored and even greater than at the unpolarized state (Fig. 1B). This demonstrates that Kv1.4 channels change localization twice during polarization of MDCK cells, a phenomenon also observed for the potassium channel Kv7.1.

Several kinases are known to be activated upon initiation of MDCK cell polarization. It is well established that PKC is activated and important for the formation of junctional complexes [32,33]. In addition, AMP-activated protein kinase (AMPK) is also strongly activated during initial epithelial cell polarization [33,34]. We have previously demonstrated that these kinases are responsible for the endocytosis of Kv1.5 and Kv7.1 during a similar calcium switch assay [15,16,24]. Based on our initial results, we therefore investigated whether pharmacological activation of endogenous PKC and AMPK would lead to removal of surface expressed Kv1.4. To this end, MDCK cells transiently expressing Kv1.4 were kept at the unpolarised state in low calcium medium and 100 nM PMA or 0.5 mM AICAR was added to activate PKC or AMPK, respectively. As demonstrated in Fig. 1C and D both PMA and AICAR treatment lead to a significant reduction in surface localized Kv1.4, a reduction comparable to that observed for Kv1.4 in the initial stage of the MDCK cell polarization. Together these data show that Kv1.4 surface expression can be downregulated by both AMPK and PKC kinases.

**Figure 2.**
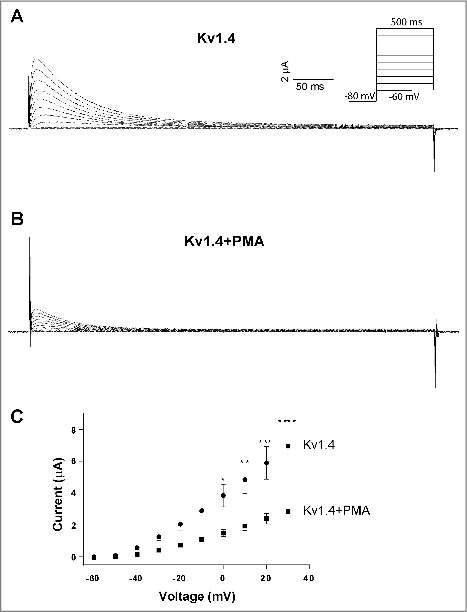
PKC activation promote reduced Kv1.4 currents. TEVC recordings of *Xenopus laevis* oocytes expressing Kv1.4 channels. Representative current traces of Kv1.4 currents following depolarization steps in control oocytes (A) and from oocytes treated with 100 nM of the PKC activator PMA (B). Insert: Step protocol. (C) Current-voltage relationship of Kv1.4 currents from control and PMA-treated oocytes. PMA significantly down-regulated the current level by 59%. Kv1.4 (n = 10) and Kv1.4+PMA (n = 7).

**Figure 3.**
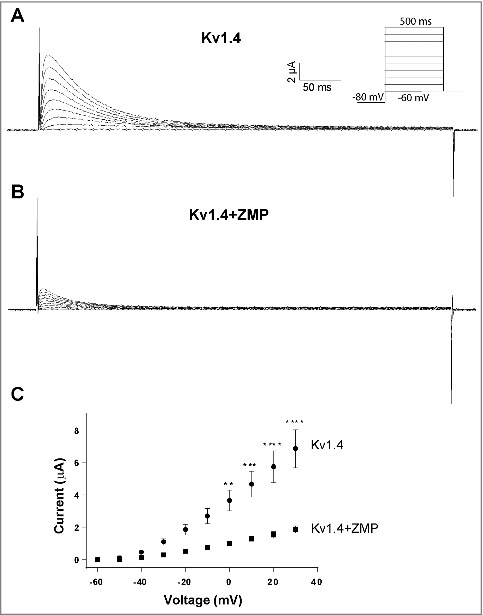
AMPK activation promote reduced Kv1.4 currents.TEVC recordings of *Xenopus laevis* oocytes expressing Kv1.4 channels. Representative current traces of Kv1.4 current following depolarization steps in control oocytes (A) and from oocytes injected with 100 ng ZMP to activate AMPK (B). Insert: Step protocol. (C) Current-voltage relationship of Kv1.4 currents in control oocytes or oocytes injected with ZMP. ZMP significantly down-regulated the current level by 73%. Kv1.4 (n = 10) and Kv1.4+ZMP (n = 7).

To verify PKC and AMPK-mediated inhibition of Kv1.4 surface expression, we expressed Kv1.4 in *Xenopus laevis* oocytes to perform two-electrode voltage clamp (TEVC) recordings. cRNA injections into *Xenopus laevis* oocytes makes it possible to control the exact amount and ratio of the different components expressed. Further, with electrophysiological recordings of *Xenopus laevis* oocytes it is possible accurately to validate the electrophysiological effects of Kv1.4 channels following the different interventions. We first applied 100 nM PMA to activate PKC for 30–45 minutes and then measured the Kv1.4 current (Fig. 2). The Kv1.4 current is a classical A-current showing fast activation followed by a relatively fast and profound inactivation when the membrane potential is depolarized [35]. Activation of PKC by application of PMA resulted in a 59% reduction in current peak amplitude at +30 mV (Fig. 2), which is in agreement with previously published observations [9]. Of note, PKC activation did not result in changed Kv1.4 current kinetics (compare current traces in Fig. 2A and B). Next, we activated AMPK in Kv1.4 expressing *Xenopus laevis* oocytes by microinjection of ZMP and measured the Kv1.4 current 30–45 min after injection. ZMP is an AMP analog, which activates AMPK by increasing the AMP/ATP ratio [36]. ZMP has previously been used to successfully activate AMPK in oocytes [15,24]. Activation of AMPK led to 73% decrease in measured Kv1.4 current peak amplitude at +30 mV (Fig. 3) thereby confirming AMPK-mediated downregulation of Kv1.4. As for PKC, AMPK activation did not alter Kv1.4 current kinetics (compare current traces in Fig. 3A and B).

**Figure 4.**
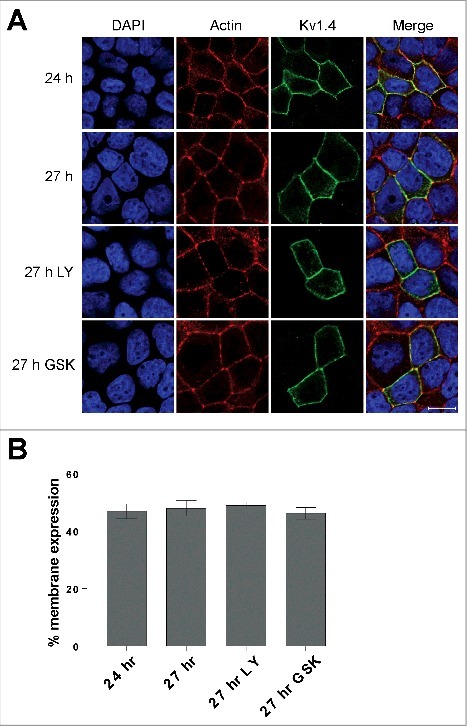
Inhibition of PI3K and SGK1 kinases do not affect Kv1.4 localization in polarized MDCK cells. (A) MDCK cells transiently expressing Kv1.4 were subjected to a calcium switch assay for 24 hr and following treated with inhibitors of either PI3K (10 µM LY-294.002) or SGK1 (1 µM GSK-650394) for 3 hr before fixation. Scalebar, 10 µm. (B) Quantification of Kv1.4 signals from (A). The Kv1.4 fluorescent cell surface Kv1.4 signal is expressed as a percentage of fluorescent Kv1.4 total cell signals. All groups were compared to the signal at 24 hr. Quantification was performed as described in the Materials and Methods section. 6 ≤ n ≤ 10 for each group. Bars represent means of each group ± SEM.

**Figure 5.**
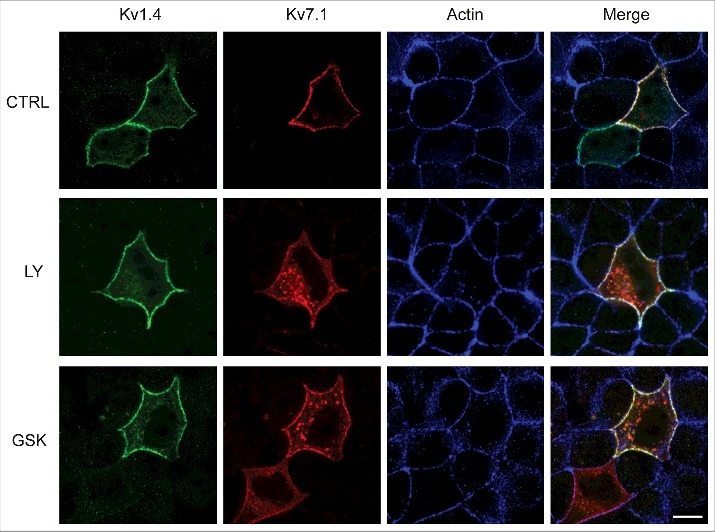
Inhibition of PI3K and SGK1 kinases selectively impacts Kv7.1 localization in polarized MDCK cells. MDCK cells transiently co-expressing Kv1.4 and Kv7.1 were treated with inhibitors of either PI3K or SGK1 for 3 hours before fixation. No effect of the PI3K inhibitor LY-294.002 (10 µM) or the SGK1 inhibitor GSK-650394 (1 µM) was observed upon the subcellular localization of Kv1.4, while Kv7.1 was endocytosed upon treatment with both. Scalebar, 10 µm.

PI3K is known to be activated during the later stages of the epithelial polarization process and we have previously reported that a PI3K-SGK1 pathway promotes surface expression of Kv7.1 by inhibiting the Nedd4-2 dependent endocytosis of the channel both during the polarization process and in fully polarized cells [14,37]. We therefore examined whether the activities of PI3K and its downstream target SGK1 had significant impact upon Kv1.4 surface expression. MDCK cells were allowed to polarize for 24 hours in order to establish the basolateral and apical membrane domains and then treated with a PI3K (LY294002, 10 µM) or an SGK1 (GSK650394, 1 µM) specific inhibitor for 3 hours before fixation. Control cells were fixed after both 24 and 27 hours. As demonstrated in Fig. 4, these treatments did not change the subcellular localization of Kv1.4. Kv1.4 was still primarily localized to the plasma membrane. Quantification of the plasma membrane associated Kv1.4 signals confirmed this observation as we found no significant difference between the inhibitor treated cells and the control cells (24 hr) (Fig. 4B). To further confirm these observations, we performed the same experiments again in MDCK cells co-expressing Kv1.4 and Kv7.1, the latter serving as a control as Kv7.1 is known to be regulated by both PI3K and SGK1 [14]. As expected, upon PI3K and SGK1 inhibition endocytosis of the Kv7.1 channels was observed, while Kv1.4 proteins remained in the surface membrane (Fig. 5).

**Figure 6.**
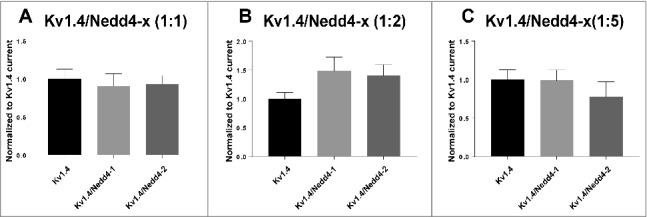
Co-expression of Kv1.4 channels with different ratios of Nedd4-x. TEVC recordings of *Xenopus laevis* oocytes expressing Kv1.4 channels with either Nedd4-1 or Nedd4-2. The current levels were measured at peak amplitude at the +30 mV pulse and normalized to the peak current for Kv1.4 expressed alone. (A) Kv1.4 and Nedd4-1 or Nedd4-2 in a molar ratio of 1:1. (B) Kv1.4 and Nedd4-1 or Nedd4-2 in a molar ratio of 1:2. (C) Kv1.4 and Nedd4-1 or Nedd4-2 in a molar ratio of 1:5. 7 ≤ n ≤ 17 for each group. Noteworthy, no change was observed on current kinetics following Nedd4-x co-expression (data not shown).

**Figure 7.**
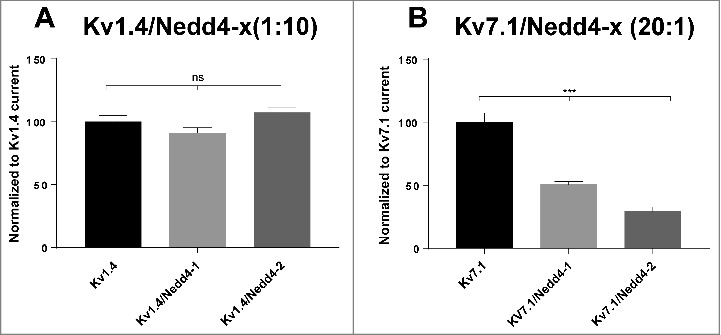
Two-electrode voltage clamp recordings of *Xenopus laevis* oocytes expressing Kv1.4 channels and Nedd4-x. The current levels were measured at peak amplitude at +30 mV for Kv1.4 and +60 mV for Kv7.1 and normalized to the peak current for Kv1.4 or Kv7.1 expressed alone. (A) Kv1.4 and Nedd4-1 or Nedd4-2 in a molar ratio of 1:10. Nedd4-x had no significant effect on Kv1.4 current levels when comparing oocytes co-expressing Kv1.4 and Nedd4-x to oocytes only expressing Kv1.4 (B) Kv7.1 and Nedd4-1 or Nedd4-2 in a molar ratio of 20:1. Nedd4-x had significant effects on Kv7.1 current levels when comparing oocytes co-expressing Kv7.1 and Nedd4-x to oocytes only expressing Kv7.1. Presented is a summary of three individual two-electrode voltage clamp experiments. 16 ≤ n ≤ 27 for each group. Noteworthy, no change was observed on current kinetics of either Kv1.4 or Kv7.1 following Nedd4-x co-expression (data not shown).

The kinase-mediated regulation of Kv1.5, Kv7.1 and Kv11.1 cell surface expression often relies on the activity of the Nedd4-2 ubiquitylating enzyme [23,38,39]. To investigate if either Nedd4-2 or the related Nedd4-1 ubiquitin ligase regulates Kv1.4, we performed recordings in *Xenopus laevis* oocytes expressing Kv1.4 with or without Nedd4-1 and Nedd4-2 (Fig. 6 and 7). Co-expression with Nedd4-1 or Nedd4-2 in different molar ratios (1:1, 1:2, 1:5, 1:10 respectively) were without significant impact on current amplitudes indicating that, unlike Kv1.5, Kv7.1 and Kv11.1, Kv1.4 is not a target of these ubiquitin ligases. As a control co-expression of Kv7.1 with Nedd4-1 or Nedd4-2 in a 20:1 ratio was performed in parallel. Even at this concentration, Nedd4 ubiquitin ligases had a drastic impact on Kv7.1 current amplitudes (Fig. 7B) in agreement with previous observations [15].

## Discussion

In the current study, we observed that initiation of MDCK cell polarization leads to a reduction in Kv1.4 channel expression at the cell surface. We have recently demonstrated similar endocytosis for the two related potassium channels Kv7.1 and Kv1.5 in response to MDCK cell polarization [15,16,24]. In these studies we found that the channels were internalized through activation of a PKC-AMPK-Nedd4-2 pathway [15,24]. Similarly, we here observe that a reduction in Kv1.4 membrane expression could be induced by stimulating either of the two kinases PKC or AMPK. In contrast, we found no effect upon Kv1.4 cell surface expression when pharmacologically inhibiting the PI3K-SGK1 pathway known to promote the surface expression of Kv7.1 in polarized MDCK cells [14]. As the PI3K-SGK1-mediated stimulation of Kv7.1 surface expression involves inhibition of Nedd4-2-mediated downregulation of Kv7.1, the absence of PI3K-SGK1 effects upon Kv1.4 could suggest that this channel is not regulated by Nedd4-2. Indeed, we found that neither Nedd4-1 nor Nedd4-2 expression had an effect on Kv1.4 current levels in *Xenopus* oocytes. This strongly suggests that the Nedd4-x family does not influence Kv1.4 activity. Together our data indicate that Kv1.4 cell surface expression and thereby activity is modulated by the two kinases PKC and AMPK. While it is possible that the two kinases can modulate the activity of Kv1.4 independently of each other e.g. by phosphorylation, it is also known that PKC can activate AMPK though the kinase LKB1 [34,40]. It is therefore possible that the observed effect of PKC activation on Kv1.4 is due to a downstream activation of AMPK. The AMPK kinase is known to play a vital role in ischemia-reperfusion injury by regulating the metabolism and cellular processes [41]. During ischemia AMPK upregulates GLUT4 in the plasma membrane to promote glucose uptake [41]. This happens through a PKC dependent mechanism [42], which could indicate that a PKC-AMPK pathway is of physiological importance in cardiac cells. If activation of a PKC-AMPK pathway downregulates Kv1.4 in cardiomyocytes following ischemia, an impact on cardiac electrophysiology would be expected. A reduction in Kv1.4 current could lead to a prolonged action potential duration, potentially opposing the abbreviating effects of I_K,ATP_ channel activation during ischemia and hypoxia [43].

Alterations in the transient outward potassium current in the early phase of action potential repolarization, where the Kv1.4 current plays a functional role, would likely increase the risk of possibly life-threatening cardiac arrhythmias [1]. Studying the underlying molecular mechanisms that are involved in the dynamic regulation of Kv1.4 channels both under physiological and pathophysiological conditions are therefor of high relevance and further studies within this field are necessary in order to gain a better understanding.

## Conclusion

Cell surface expression regulation through extensive kinase-mediated pathways is evident for a number of voltage-gated potassium (Kv) channels important in cardiac electrophysiology. In this study we investigated the mechanisms of the Kv1.4 (I_to,s_) channel regulation through kinase activation. We here report that both PKC and AMPK activation leads to reduction in Kv1.4 current levels. This reduction most likely happens though Kv1.4 endocytosis. The mechanisms governing this internalization are still not fully elucidated; however, our data indicate that this does not involve the Nedd4-x family, which is known to be involved in similar endocytic responses of highly related Kv potassium channels.

## Materials and methods

### Antibodies and chemicals

The antibodies used were: mouse monoclonal anti-Kv1.4 (1:50, clone K13/31, UC Davis/NIH NeuroMab Facility), goat polyclonal anti-Kv7.1 (1:100), Alexa Fluor®488-conjugated donkey anti-mouse IgG (1:200) and Alexa Fluor®555-conjugated donkey anti-goat IgG (1:500). Alexa Flour®647 phalloidin (1:200) and 4′,6-diamidino-2-phenylindole (DAPI) was used to stain actin filaments and nuclei, respectively. All fluorescent antibodies and probes were purchased from Thermo Fischer Scientific.

The activators and inhibitors used in this study were as follows: 5-aminoimidazole-4-carboxamide-1-β-D-ribofuranoside (AICAR, Sigma-Aldrich, 0.5 mM), 5-aminoimidazole-4-carboxamide-1-β-d-ribofuranosyl-5′-monophosphate (ZMP, Sigma-Aldrich), 2-O-Tetradecanoylphorbol 13-acetate (PMA, Sigma-Aldrich, 100 nM), 52-(4-Morpholinyl)-8-phenyl-1(4H)-benzopyran-4-one hydrochloride (LY294002, Sigma-Aldrich, 10 µM), 2-Cyclopentyl-4-(5-phenyl-1*H*-pyrrolo[2,3-*b*]pyridin-3-yl-benzoic acid (GSK650394, Tocris Bioscience, 1 µM).

### Transient expression in MDCK cells

MDCK (strain II) cells [44] were grown in DMEM (Thermo Fischer Scientific) supplemented with 100 U/ml penicillin, 100 mg/ml streptomycin and 10% FBS (Sigma-Aldrich) at 37°C in a humidified atmosphere with 5% CO_2_.

MDCK cells were transfected in suspension with 2 µg of pXOOM-rKv1.4 [45] or 2 µg pXOOM-rKv1.4 and 0.5 µg pXOOM-hKv7.1 (PMID: 12096056) using Lipofectamine and Plus Reagent (Thermo Fischer Scientific) according to manufacturer's protocol. After the mixing of cells and transfection reagents, the mixture was added to 35 mm dishes containing glass coverslips (12 mm in diameter, VWR) and the cells were allowed to attach to the coverslips during the transfection procedure. 3 hours after initiation of transfection, the transfection medium was changed to DMEM medium.

### Calcium switch experiment

Transiently transfected MDCK cells plated on glass coverslips were subjected to a calcium switch experiment as previously described [16]. Briefly explained, MDCK cells were grown in a low calcium medium (calcium-free S-MEM (Sigma-Aldrich) supplemented with 10% dialyzed fetal bovine serum (Thermo Fischer Scientific), 100 U/mL penicillin, 100 mg/ml streptomycin, 2 mM L-glutamine and 1,6 μM calcium chloride) until reaching confluency (approximately 2 days). The medium was then changed back to DMEM medium, which initiated the polarization process.

### Immunofluorescence

MDCK cells grown on glass coverslips were fixed in 4% paraformaldehyde in PBS for 30 minutes at room temperature. Blocking of unspecific binding was performed by a 30-minute incubation with 0.2% fish skin gelatin in PBS supplemented with 0.1% Triton X-100 (PBST). The cells were incubated for 1 hour in primary antibody diluted in PBST. Secondary antibody and fluorescent probes were diluted in PBST and applied for 45 minutes. The coverslips were mounted in Prolong Gold or Prolong Diamond (Thermo Fischer Scientific).

### Confocal microscopy and imaging

Laser scanning confocal microscopy was performed using the Leica TCS SP2 system equipped with argon and helium-neon lasers or a Zeiss LSM 710 confocal system. Images were acquired using a 63x water immersion objective (NA 1.2) for Leica TCS SP2 or a 63x oil immersion objective (NA 1.4) for Zeiss LSM 710. Pinhole size was 1 AU and the pixel format 1024 × 1024. Sequential scanning was employed to allow the separation of signals from the individual channels and line averaging was used to reduce noise. Acquired images were treated using the Zen 2011 blue edition confocal software (Carl Zeiss, Germany) or Adobe Photoshop CS6.

### Quantifications of membrane expression

Fluorescent signals were quantified using the Zen 2011 blue edition confocal software as previously described [16]. In short, the localization of the plasma membrane was defined by plasma membrane-associated Alexa®Fluor 647 phalloidin staining. Regions were drawn on either side of this staining to obtain the intensities of the fluorescent signals originating from the entire cell or the cell interior. For data analysis, signals originating from the surface membrane were obtained by subtracting the intracellular signal from the total cell fluorescent signal. A total of 10 > n > 6 cells from 3 independent experiments were quantified for each condition.

### mRNA generation and oocyte injection

mRNA was generated from linearized pXOOM plasmids using the Ambion T7 mMessage mMachine kit (Ambion, Austin, TX, USA) according to manufacturer's instructions. The mRNA concentrations were determined using a ND-1000 NanoDrop UV spectrophotometer, and mRNA integrity was confirmed by gel electrophoresis. mRNA was stored at −80°C until injection.

*Xenopus laevis* oocytes were purchased from EcoCyte Bioscience, Germany. Upon delivery, they were kept at 19°C for 24 hours in Kulori solution (4 mM KCl, 90 mM NaCl, 1 mM MgCl_2_, 1 mM CaCl_2_, 5 mM HEPES, pH 7.4). Subsequently, they were injected with 50 nl of Kv1.4 mRNA (50 pg) using a Nanoject microinjector (Drummond Scientific, Broomall, PA, USA). For co-expression of Kv1.4 and Nedd4-1 or Nedd4-2, the mRNAs were mixed in molar ratios of 1:1, 1:2, 1:5 and 1:10 prior to oocyte injection. For measurements on Kv7.1, 10 ng of Kv7.1 mRNA was injected alone or in combination with Nedd4-1/Nedd4-2 in a 20:1 molar ratio. For optimal expression conditions, the oocytes were kept in Kulori medium at 19°C for 48–72 hours before functional measurements were performed.

### Electrophysiology

Kv1.4 currents in *Xenopus laevis* oocytes were measured using the two-electrode voltage-clamp technique at room temperature under continuous superfusion with Kulori-buffer (6 ml/min). Oocytes were impaled with a current electrode and a voltage clamp electrode pulled from borocilicate glass capillaries (Sutter Instrument Co. model P-97 Puller). When filled with 3 M KCl both electrodes had a tip resistance between 0.5–2.5 MΩ. The condition of each single oocyte was tested before measurements by recording the membrane potentials. Oocytes with endogenous resting membrane potential more positive than −35 mV and with leak current above 1000 nA were discarded. The data was recorded using a Dagan CA1B amplifier (Minneapolis, MN, USA), a HEKA EPC-9 interface and HEKA Pulse v2 × 73.2 software (HEKA Elektronik, Lambrecht/Pfalz, Germany). The currents were analyzed by applying a standard step protocol with 500 ms long pulses from −60 to +30 mV with 10 mV increments from a holding potential of −80 mV.

For the PMA experiments oocytes were treated with vehicle or 100 nM PMA and for the ZMP experiments oocytes were injected with vehicle or 100 ng ZMP. Current recordings were conducted between 30 and 45 minutes after the oocytes treatment or injection.

Kv7.1 currents were analyzed by applying a standard step protocol with pulses from −100 to +60 mV (3 s) in 20 mV increments from a holding potential of −80 mV. The tail currents were measured at −30 mV (1s).

### Data and statistical analyses

All data are presented as mean ± standard error of mean (SEM).

For the confocal data, one-way ANOVA and Dunnett's multiple comparisons test were used to compare the mean values of % membrane expression at the unpolarized state with different time-points upon initiation of the polarization process. The same tests were further used for the comparison of % membrane expression before and after treatment with the kinase activator and inhibitors (Fig. 1B and 1D).

The statistical analysis of the electrophysiological data was performed with two-way ANOVA and Sidak's multiple comparisons test to compare mean values of the obtained current levels (µA) (Fig. 2 and 3).

P-values < 0.05 were considered to be statistically different. PatchMaster (v2 × 90 HEKA Elektronik), Igor Pro 4.04 (Wavemetrics, USA) and GraphPad Prism 7 (GraphPad Software, USA) were used for data and statistical analysis. The statistical significance in figures is denoted by * = P < 0.05, ** = P < 0.01, *** = P < 0.001 and **** = P < 0.0001.
